# Incidence, Management and Outcomes in Women Undergoing Peripartum Hysterectomy in a Tertiary Care Centre in India

**DOI:** 10.7759/cureus.14171

**Published:** 2021-03-29

**Authors:** Vidhi Chaudhary, Meenakshi Singh, Shilpi Nain, FNU Reena, Kiran Aggarwal, Ratna Biswas, Manju Puri, Janithya Pujari

**Affiliations:** 1 Department of Obstetrics and Gynaecology, Lady Hardinge Medical College, Delhi, IND

**Keywords:** peripartum hysterectomy, morbid adherent placenta, postpartum haemorrhage, blood transfusion, placenta previa major, previous caesarean

## Abstract

Background

Peripartum hysterectomy (PRH) is the surgical removal of the uterus performed in obstetrical complications such as uncontrolled postpartum haemorrhage (PPH), unrepairable uterine rupture, and sepsis. Its incidence has increased in recent years. The objective of this study was to review all the cases of PRH in a tertiary care teaching hospital over three years (January 2017-December 2019) to determine its incidence and analyse clinico-demographic characteristics in these women.

Method

All women undergoing PRH from January 2017 to December 2019 were included in the study. Data were collected retrospectively from medical records, of patients who underwent a PRH at the time of delivery, or within 24 hours, or performed any time before discharge from the same hospitalization and obstetric event. The total number of deliveries including caesarean and vaginal deliveries were recorded. Main outcome measures were the incidence of PRH, indication for hysterectomy, management option used, maternal outcomes (PPH, bladder injury and maternal death) and fetal outcomes (stillbirth).

Results

There were a total of 3904,4 deliveries; 27,337 vaginal and 11,697 caesarean sections in three years. A total of 50 patients underwent a PRH. The incidence of PRH in our study was 1.3 per 1,000 deliveries and 3.5/1,000 caesareans, respectively. PRH was found to be more common following cesarean sections than vaginal deliveries (odds ratio 22.86 [95% CI: 8.16 to 63.98]). Morbid adherent placenta (MAP) (n=30, 62%) was the most common indications of PRH. Seven (15%) women had PRH due to uterine rupture. Twenty-seven women of the 30 women (90%) with the MAP had a previous caesarean delivery. The case fatality rate per hysterectomy was 4%. Stillbirth rate (SBR: n=8,16%) among women having PRH was seven-fold higher than overall SBR in our country.

Conclusion

There has been a rise in MAP as an indication of PRH in our study for a decade in comparison to uterine atony. Caesarean delivery is a significant risk factor for PRH. Previous caesarean section and major placenta previa were common occurring obstetric risk factors present in the MAP in our cohort. Our maternal mortality in PRH was low and the stillbirth rate was high when compared with national data.

## Introduction

Peripartum hysterectomy (PRH) is a surgical procedure performed at the time of delivery or in the immediate postpartum period, as a last resort in the treatment for severe postpartum haemorrhage (PPH), morbid adherent placenta (MAP), uterine rupture or genital sepsis and is associated with high maternal morbidity and mortality [1,2]. The Maternal Mortality Ratio of India has declined from 178 in 2010-12 to 113 in 2016-18 [3]. This has been consequent to an increase in institutional deliveries, timely management of obstetrical haemorrhage and early use of PRH for haemorrhage [3,4]. However, this has led to an increase in PRH in recent years [5-7]. To understand the magnitude of the problem, we reviewed our data retrospectively with an aim to determine the incidence and analyse indications, management, maternal and foetal outcomes in women who underwent PRH in our centre.

## Materials and methods

Study population and case definition

This was a descriptive, retrospective cohort study in which all women undergoing PRH from January 2017 to December 2019 in the Department of Obstetrics and Gynaecology at Lady Hardinge Medical College, New Delhi, India were identified from the medical records. The records were reviewed in detail and data was collected to meet our objectives. The total number of deliveries including cesarean and vaginal deliveries in the study period were also recorded.

PRH was defined as hysterectomy performed at the time, or within 24 hours, or at any time from delivery beyond 20 weeks of gestation to discharge from the primary obstetric event. Primary PRH was defined as hysterectomy undertaken within 24 hours of delivery whereas secondary or delayed PRH was defined as hysterectomy undertaken after 24 hours from the index obstetric event.

Data collection and grouping of data

All cases were coded to minimize patient identification. All cases conforming to the case deﬁnition were included in the study. Relevant demographic and clinical data (such as age, parity, type of delivery, indication for hysterectomy, obstetric risk factors, management, and outcomes) were recorded and analyzed. Blood loss was estimated by direct estimation of blood collected in calibrated suction apparatus, number of lap pads fully soaked with blood (pre-estimated as 60 ml of blood loss) and measuring blood collected via drapes and was recorded in case sheet. Preoperative obstetric ultrasound including morphology, location of the placenta and operative notes, and histopathology of the uterus and placenta were used to confirm the final diagnosis.

Statistical analysis

Data were coded and recorded in the MS Excel spreadsheet program. SPSS v23 (IBM Corp., Armonk, NY) was used for data analysis. Descriptive statistics were elaborated in the form of the mean (95% CI: confidence interval)/standard deviations and medians (interquartile range) IQRs, for continuous variables, and frequencies and percentages for categorical variables. The odds ratio was calculated, wherever applicable.

## Results

A total of 50 patients underwent PRH as per case definition in the period of three years in our hospital. The total number of deliveries during this period was 39,044 with 27,337 vaginal deliveries and 11697 caesarean deliveries. There was a total of four hysterotomies and six laparotomies in this period. These are the true denominators as all deliveries are recorded into the central database of the medical records section. There were five hysterectomies which followed one hysterotomy done in view of placenta previa with intractable antepartum hemorrhage at 22 weeks and four laparotomies done due to uterine rupture. Caesarean deliveries for calculating incidence consist of all CS done above 20 weeks of gestation due to obstetric indications. The incidence of PRH in our study was 0.14/1,000 vaginal deliveries and 3.5/1,000 caesarean (CS) deliveries. Hence the overall incidence of PRH was 1.3 per 1,000 deliveries. We included 48 cases of PRH for our final detailed review as two had incomplete data, hence were excluded from the study (Table 1).

**Table 1:** Incidence of peripartum hysterectomy (PRH) by deliveries in study period from January 2017 to December 2019.

	N (%)	Incidence per 1,000 deliveries
Total deliveries	39,044	-
Total vaginal deliveries	27,337 (70%)	-
Total caesarean deliveries	11,697(29.9%)	-
Total PRH cases	50 (0.13%)	1.3
PRH following vaginal deliveries	4 (0.014%)	0.14
PRH following caesarean sections	41 (0.35%)	3.5

Demographics and clinical characteristics

The mean age of women undergoing hysterectomy was 29.46 years, with 39 women (69%) less than 30 years. Thirty women (63 %) were unbooked (presented to the hospital for the first time in their current pregnancy). The primary mode of delivery in women who subsequently had hysterectomy was caesarean section (n=39.81%). Caesarean delivery was associated with higher odds of having PRH [22.86 (95% CI: 8.16 to 63.98)] when compared with vaginal deliveries.

Thirty women (62%) were anaemic with mean preoperative haemoglobin of 9.64 g/dl. Booking status in PRH was commonly associated with anaemia (n=7/30.39% booked vs n=23/30.77% unbooked). Sixty-six percent of women underwent emergency hysterectomy when compared with planned procedure (Table 2).

**Table 2:** Demographic characteristics.

	n (%)	Mean ± SD (range)
Booking status		
Booked	18 (37.5%)	
Unbooked	30 (62.5%)	
Age (years)		29.46±4.51 (22.00-40.00)
Age		
21-30 years	33 (68.8%)	
31-40 years	15 (31.2%)	
Parity		1.68±0.68 (0-5)
P0	2 (4.2%)	
P1	15 (31.2%)	
P2	26 (54.2%)	
P3	4 (8.3%)	
P5	1 (2.1%)	
Parity status		
Nullipara	2 (4.2%)	
P1	15 (31.2%)	
≥P2	31 (64.6%)	
Previous curettage (present)	15 (31.2%)	
Previous caesarean delivery	36 (75.0%)	
Previous vaginal delivery	15 (31.2%)	
Mean gestational age at current delivery (weeks)		34.94±3.94 (22.00-41.00)
Mean gestational age at current delivery (weeks)		
<37 weeks	31 (64.6%)	
≥37 weeks	17 (35.4%)	

Commonly prevalent obstetric risk factors for PRH in our study were previous caesarean section and major placenta previa. Total 30 women (62.5%) had placenta previa in index pregnancy of which 26 (86.6%) were morbid adherent.

Morbid adherent placenta (MAP; 30/48; 62.5%) and intractable hemorrhage (11/48;22.9%) were major indications for PRH. Of all the women with intractable hemorrhage, eight had atonic PPH, the other two women had more than two factors of PPH (one with atonic and coagulopathy and another with atonic and adherent placenta) and one woman had a secondary hemorrhage on day 7 of primary caesarean due to sepsis and uterine necrosis. Uterine rupture and uterine sepsis were other indications of PRH. Seven women (15%) had a uterine rupture. Of these four (8%) had ruptured at arrival in our emergency room, two (4%) women had rupture following delivery after trial of vaginal birth after caesarean (VBAC) and one had (2%) perioperative partial uterine rupture during caesarean. This woman was given a trial of a vaginal birth but developed acute fetal bradycardia for which she had emergency CS. Four (8%) women had a hysterectomy for uterine sepsis and necrosis. In four patients there was more than one indication, uterine sepsis and secondary intractable hemorrhage in two, morbid adherent placenta with uterine rupture in one, and intractable hemorrhage with the morbid adherent placenta in one.

Causes and management of associated haemorrhage

**Figure 1:**
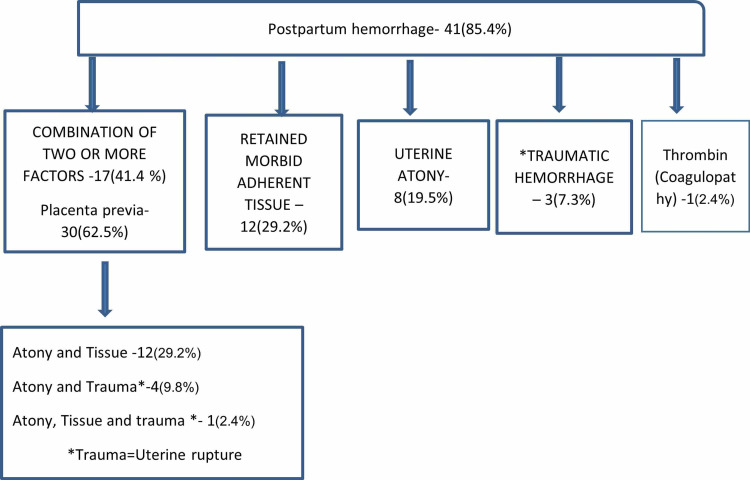
Etiology of postpartum hemorrhage in peripartum hysterectomy.

Major haemorrhage (41/48, 85%) was the most common intraoperative complication in women undergoing PRH. Almost half of the women (17/41, 41.2%) had hemorrhage due to two or more factors (uterine atony, morbid adherent placenta, and traumatic uterine rupture). Uterine atony alone was present in one-fifth of women (Figure 1).

Blood loss was more in women with placenta previa major as a risk factor and hysterectomies were undertaken because of the morbid adherent placenta. There was significantly higher mean blood loss in hysterectomies undertaken in view of morbid adherent placenta when compared with other indications (4.02±1.56 liters vs 3.01±1.39 liters; p-value = 0.025, student t-test) (Tables 3, 4).

**Table 3:** Clinical characteristics. *Four women had two or more causes, so total exceeds -100%.

Clinical characteristics	n (%)
Obstetric risk factor	40 (83%)
Placenta previa major	30 (62.5%)
Hypertensive disorder of pregnancy	6 (12.5%)
Others – breech, abruptio, Multiple pregnancy	4 (8%)
Primary mode of delivery	
Caesarean section	39 (81.2%)
Laparotomy	4 (8.3%)
Normal vaginal delivery	4 (8.3%)
Hysterotomy	1 (2.1%)
Indication for hysterectomy*	
Morbid adherent placenta	30 (62.5%)
Intractable haemorrhage	11 (22.9%)
Uterine rupture	7 (14.6%)
Uterine sepsis and necrosis	4 (8.3%)
Timing of hysterectomy	
Primary	44 (92%)
Secondary	4 (8%)
Type of hysterectomy	
Total	40(83.3%)
Subtotal	8(16.4%)

**Table 4:** Subgroup analysis: clinico-demographic characteristics in PRH by morbid adherent placenta. *Non-morbid adherent placenta cases include intractable hemorrhage, uterine rupture, and sepsis as an indication of PRH. PRH: peripartum hysterectomy; NICU: neonatal intensive care unit; CS: caesarean section; LSCS: lower segment caesarean section; NVD: normal vaginal delivery.

Study variables	Morbid adherent placenta, n=30, n%, Mean	Non-morbid adherent placenta*, n=18, n%, Mean
Age (years)		
21-30 years	19 (65.5%)	14 (73.7%)
31-40 years	10 (34.5%)	5 (26.3%)
Parity status		
Nulliparous	1 (3%)	1 (5.3%)
Multiparous	29 (97%)	17 (94%)
Previous caesarean delivery	27 (90%)	9 (50%)
Mean gestational age at current delivery (weeks)		
<37 weeks	24 (80.0%)	10 (55.6%)
≥37 weeks	6 (20.0%)	11 (61.1%)
Placenta previa major	26 (86.7%)	4 (22.2%)
Primary mode of delivery		
Caesarean section	28 (93.3%)	11 (61.1%)
Laparotomy	1 (3.3%)	3 (16.7%)
NVD	1 (3.3%)	3 (16.7%)
Type of uterine incision		
LSCS	11 (36.7%)	11 (61.1%)
Classical CS	18 (60.0%)	0 (0.0%)
Uterotonics (required)	26 (86.7%)	15 (83.3%)
Internal iliac artery ligation (required)	28 (93.3%)	6 (33.3%)
Blood loss (L)	4.02 ± 1.56	3.01 ± 1.39
Packed cell transfused	5.60 ± 2.44	4.22 ± 1.52
Bladder injury and repair (present)	4 (13.3%)	1 (5.6%)
NICU admission (required)	7 (23.3%)	0 (0%)
Birth weight (kg)	2.07 ± 0.71	2.63 ± 0.92
Stillbirth	2 (25.0%)	6(75%)

Uterotonics (the combination of oxytocin, prostaglandins, and tranexamic acid) were administered in 41 women (85%) including two cases of secondary hysterectomy which were done in view of sepsis and associated secondary haemorrhage. Seven women (16%) did not receive any therapy for haemorrhage and were solely treated by immediate hysterectomy. These women had less blood loss and less requirement of fresh frozen plasma compared to those requiring uterotonics. Uterotonic requirements were high in those with an increased amount of blood loss (3.94±1.43 liters vs 1.91±1.18 litres). Descriptive data regarding the management of hemorrhage is given in Table 5. All women received broad-spectrum antibiotics after the PRH. One of the women requiring compression sutures for PPH developed uterine necrosis with sepsis unresponsive to antibiotics following the use of the compression sutures and had secondary hysterectomy [8].

**Table 5:** Management and outcomes of PRH. PRH: peripartum hysterectomy; ICU: intensive care unit; NICU: neonatal intensive care unit; FFP: fresh frozen plasma.

Study variables	n (%)	Mean ± SD (range)
Compression sutures in primary caesarean (required)	3 (6.2%)	
Uterotonics (required)	41 (85.4%)	
Uterine artery ligation (required)	16 (33.3%)	
Internal iliac artery ligation (required)	34 (70.8%)	
Anaesthesia		
General anaesthesia (GA)	40 (83.3%)	
Subarachnoid block (SAB)+GA	7 (14.6%)	
SAB	1 (2.1%)	
Duration of Surgery (hours)		3.44±0.73
Blood loss (Litres-L)		3.64±1.56
Packed cell transfused units		5.08±2.23
FFP transfused units		4.96±3.20
Mechanical ventilation (required)	38 (79.2%)	
Days of mechanical ventilation		1.63±2.15
ICU admission (required)	47 (97.9%)	
Days of ICU stay		2.43±4.27
Adverse maternal outcomes		
Febrile illness	9 (18.7%)	
Lower respiratory infection and pneumonia	7 (14.6%)	
Wound sepsis	7 (14.6%)	
Urinary tract infection	6 (12.5%)	
Genital sepsis	5 (10.4%)	
Bladder injury and repair	5 (10.4%)	
Resuturing of wound	3 (6.2%)	
Hypoxic seizures	1 (2.1%)	
Maternal death	2 (4.2%)	
Fetal outcomes		
Live births	40 (81.6%)	
Stillbirth	8 (16.3%)	
Abortus	1 (2%)	
Birth weight (kg)		2.28±0.83
NICU admission (required)	8 (16.3%)	

Maternal and fetal outcomes

Total adverse maternal and fetal outcomes due to PRH were in 27 (56%) women and in 28 babies (58%). The febrile illness was the most common post-PRH complication and the Bladder injury was the second most common intraoperative complication. Lower respiratory tract infection and pneumonia were positively associated with a higher amount of blood loss (4.39±0.90 vs 3.51±1.63 liters). Two women died, one due to coagulopathy, and the other had uterine sepsis. The maternal mortality ratio for PRH was 5 per 100,000 live births. Stillbirth rate was 16% (Table 5).

## Discussion

This study is a review of PRH performed in recent three years from January 2017 to December 2019 at a tertiary referral center in India. Worldwide, the rate of PRH varies widely from rates less than one in 1,000 deliveries to as high as 50/1,000 deliveries [6,9,10]. Nordic countries have an extremely low incidence of PRH of 0.35 per 1,000 births compared with 50 per 1,000 in Asia and Africa. The incidence of PRH in our hospital was 1.3 per 1,000 deliveries which is below the reported rates in Asia and India [6,11]. This has been due to close monitoring of pregnant women in labor and delivery by skilled health care providers, active management of PPH with early use of uterotonics, and stepwise devascularisation for PPH during caesarean in our population. However, the incidence has doubled since the last decade in our hospital due to an increase in the morbid adherent placenta as an indication of PRH [12].

Women who underwent PRH in our study were younger (30 years or less) compared to high-income countries where the mean age was 34.5 ± 5.5 years [9,10]. Younger age at hysterectomy can be attributed to younger age at marriage and childbearing in our country. A similar trend was also seen in the woman’s trial, where the mean age was 28 years among women in Asia and Africa undergoing PRH [6].

Our study had a fair number of unbooked women. Unbooked women were those who had either one or no antenatal visit in a designated healthcare center and presented at our hospital in labor or antepartum haemorrhage with a high-risk condition such as placenta previa, morbid adherent placenta, or uterine rupture. Five cases of uterine rupture, who presented to our hospital were unbooked, thereby missing the opportunity of early intervention for impending scar dehiscence, which could have prevented PRH. This was attributed to a lack of understanding about the possible risk in current pregnancy and perceiving pregnancy as a natural process. Unbooked status was significantly associated with anaemia due to poor nutrition and limited intake of recommended iron and folic acid tablets [13]. This remains an area of concern and has been pointed out in a national survey [14]. Routine antenatal care (ANC) plays an important role in the early identification of complications, correction of modifiable risk factors, and planning of interventions in high-risk conditions. National programs promote regular ANC care and free institutional deliveries, and its uptake has been increasing, but still, many women present in the hospital either when a complication has developed or in labor.

PRH was significantly higher when caesarean section (CS) was the primary mode of delivery compared to vaginal deliveries in our center. Three-fourths of women who had caesarean delivery had a previous CS. The incidence of CS has increased in India and worldwide [6,7,11]. Previous caesarean and caesarean delivery in an index pregnancy is a strong risk factor for emergency peripartum hysterectomy with higher risks conferred for each additional CS done [10,15,16]. In addition, the previous caesarean is a risk factor for the morbid adherent placenta [17]. Hence efforts must be directed to prevent primary CS in the first pregnancy which will have a role in reducing the PRH. 

Placenta previa major was the second common obstetric risk factor in our women undergoing PRH and 26 (86%) of these women had MAP. An abnormal vascularization resulting from the scaring process after surgery (CS or previous curettage), with secondary localized hypoxia, leads to both defective decidualization and excessive trophoblastic invasion causing previa and morbid adherence [18]. Systematic reviews propose that all patients with the previous scar must be accurately assessed for placenta previa and invasive placentation by doppler ultrasound to optimize delivery. However, preoperative diagnosis of MAP was unavailable in 13 women (43%), as these were unbooked, presented in labor in emergency hours, and required urgent delivery for obstetric indications, hence could not have an expert ultrasound diagnosis. In such situations, a high probability of MAP must be considered in presence of the previous CS and major placenta previa [19]. In our study, obstetric consultants and senior anaesthetists were available during the surgery which helped in the timely management of these women. We have a surgical policy of total PRH in presence of MAP. The placenta accreta care bundle was followed as per recommended guidelines [19]. After birth, MAP remains attached firmly to the uterine wall causing severe blood loss, and time to attempt conservative management is limited [17]. Uterine preservation methods involve leaving the placenta in situ and uterine artery embolization (UAE). Both procedures are associated with risk of sepsis, thrombosis, secondary haemorrhage and require long follow-up [19]. As embolization is not available in our hospital and there is limited compliance for long follow-up in our women, all cases of MAP were managed with hysterectomy.

Worldwide, there has been a shift in the primary indication of PRH from uterine atony to MAP [12,20]. Our study showed MAP as the most common cause and intractable hemorrhage due to uterine atony as a second most common cause of PRH [21,22]. There are variations in definitions of uterine atony in different studies [21-23]. We feel hemorrhage due to atony in MAP, is an outcome and should be included as its immediate complication. Hence atony as an indication of hysterectomy is lower in our study. Uterine rupture as a cause of hysterectomy is on the decline as was in our study (15%). It is due to improved antenatal care, early registration, and counseling in hospital and institutional delivery for women with previous caesarean [7]. 

An immediate complication in PRH was a major haemorrhage with a mean blood loss of 3.64±1.56 liter. The cause of haemorrhage was the combination of two factors or more. MAP was a major contributor to PPH. (Figure 1). There was higher mean blood loss in hysterectomies undertaken in view of morbid adherent placenta when compared with other indications (4.02±1.56 liters vs 3.01±1.39 liters). This is because the presence of adherent tissue results in improper retraction of the lower segment and atonicity of the upper segment due to the presence of adhered tissue [16,23]. This reflects a changing trend from uterine atony as the sole cause of PPH to the multifactorial nature of hemorrhage due to the presence of morbid adherent placenta [16,20].

An important aspect of managing major hemorrhage in PRH is the uninterrupted supply of sufficient units of blood and blood products. This was ensured by effective communication between haematologist, blood bank, and surgical team. PPH protocol was followed as per recommended guidelines [19]. Therapies to control haemorrhage in our study were uterotonics, tranexamic acid, compression sutures, and stepwise devascularisation of uterine or internal iliac arteries or both. Use of internal iliac artery ligation (IIL) in MAP and intractable PPH was invaluable during PRH. It helped in the control of heavy bleeding arising due to atony or adhered placental tissue and helped in better visualization and completeness of surgical procedure. For MAP, internal iliac ligation was done prior to the start of hysterectomy after the delivery of the baby. For all other cases of haemorrhage, IIL was done when bleeding was refractory to medical methods, uterine artery ligation, or compression sutures. In the future, these cases will be encountered on a frequent basis, hence all obstetricians must develop the necessary training and skill to perform IIL [21-24]. We did not use balloon tamponade due to acuteness of event (PPH), and recombinant activated factor VII due to its unavailability at our center. We, however, feel their use could have prevented PRH in cases of uterine atony not complicated by the adherent placenta. Worthwhile to note is that none of the women with uterine rupture or sepsis needed IIL. This is because hysterectomy has rapidly proceeded in unrepairable rupture and, bleeding was arrested after uterine arteries were ligated. Hemorrhage was managed in all but one case. This woman had presented with abruption placenta, with massive antepartum hemorrhage and shock for which emergency caesarean was done. PRH was done as a measure to control PPH as atonicity and coagulation failure continued despite adequate blood replacement, uterotonics, and devascularisation including IIL. This woman had mortality despite PRH, and continued resuscitative efforts.

Most women were managed postoperatively in the intensive care unit (ICU) and required mechanical ventilation. We feel that ICU care had a direct effect on hemodynamic stabilization of patients and minimizing postoperative morbidity.

One-tenth of PRH cases had bladder injury. There were no ureteric injuries that are comparable to other studies [7,16]. Eighty percent of bladder injuries(4/5) occurred in MAP and in those with the previous caesarean, making it a risk factor. However, larger prospective population studies are required to ascertain it. Previous caesarean causes adhesion formation between the bladder base and the lower uterine segment (LUS) [25]. These injuries commonly occur while separating the bladder base from LUS during caesarean or PRH. In our study, all injuries occurred perioperatively and were immediately diagnosed and repaired by the surgeon. The catheter was left in situ for 14 days. All patients recovered. Hence identifying injuries perioperatively is key in preventing long-term morbidity.

Postoperative adverse maternal events were febrile illness, urinary tract infections, genital sepsis, wound sepsis, and pneumonia, all of which responded to broad-spectrum antibiotics. Women who developed lower respiratory tract infections and pneumonia had higher units of packed cell transfusion due to significant blood loss during PRH. Liberal use of blood products can result in a risk of serious infections such as pneumonia, bloodstream infections, and wound infections [26]. Hence broad-spectrum antibiotics must be given in the event of massive transfusion and sepsis, which helps in the recovery of women. One woman had repeated hypoxic seizures. This woman was multiparous and had PRH due to massive atonic PPH. She was managed in ICU and subsequently recovered.

 Though PRH was performed as a lifesaving procedure, there were two maternal mortality. Our case fatality rate per hysterectomy was 4% (4 per 100 hysterectomies) which is comparable to few developed countries [24]. We feel our maternal mortality in PRH was low despite the presence of major haemorrhage, anaemia, and unbooked status. This was attributed to an early decision for hysterectomy, immediate blood and blood products replacement, effective liaison between experienced obstetrician, surgeon, and anesthetist in management of PRH and post-surgical care in ICU.

Few studies have mentioned fetal outcomes in PRH. There was a greater percentage of preterm delivery in women undergoing PRH. It was either due to an elective delivery undertaken in diagnosed cases of MAP earlier than 37 weeks of gestation or emergent obstetric indication such as labor or hemorrhage in MAP women necessitating delivery prior to term [19]. Neonatal intensive care unit (NICU) admission was required in babies of seven women. All belonged to women undergoing PRH due to morbid adherent placenta. However, there was no early neonatal mortality (first seven days) in these babies. This was due to steroid rescue, wherever possible, the timely decision of delivery in MAP, and the presence of a neonatologist at the time of delivery with access to NICU care if required.

The stillbirth rate was seven-fold higher in our study when compared with the overall stillbirth rate in India [27,28]. It was seen that women who had stillbirths, had low mean preoperative haemoglobin when compared with live births (8.22±2.75 gm/dl vs 10.02±1.69 gm/dl). PRH is a risk factor for stillbirth when undertaken in the presence of maternal complications such as a ruptured uterus, obstetric haemorrhage, MAP, placental abruption, and associated severe anaemia. These conditions cause fetal anoxia leading to intrauterine demise which is likely cause in our babies [29,30]. Uterine rupture as an indication of PRH in our study had higher fresh stillbirth than MAP [n=5 (62.5%) vs n=2 (25%)]. These stillbirths, we feel were preventable if these women had regular antenatal visits and had presented earlier when in labor, to our hospital.

## Conclusions

PRH in our country is high when compared with the developed world, though lower than in Asia. The most important risk factor for PRH was caesarean in the index pregnancy. Our study found an increased occurrence of previous caesarean, placenta praevia, MAP in women undergoing peripartum hysterectomy. Hence preventing primary caesarean in the first pregnancy is key in reducing the risk of peripartum hysterectomy. Another important factor was unbooked status which resulted in life-threatening morbidity of rupture of previous CS scar and stillbirth. Hence, we must focus on educating pregnant women for regular antenatal visits and monitored deliveries in the hospital so that timely identification of high-risk factors and appropriate management can be done.
